# Effects of treatment with clopidogrel with or without proton pump inhibitor omeprazole on the risk of ischemic stroke: a nationwide cohort study

**DOI:** 10.1038/s41598-024-51682-8

**Published:** 2024-01-19

**Authors:** Chao-Chien Chang, Yu-Ching Chou, Jin-Yin Chang, Chien-An Sun

**Affiliations:** 1 Division of Cardiology, Department of Internal Medicine, Cathay General Hospital, Taipei City, Taiwan; 2 Graduate Institute of Medical Sciences, College of Medicine, Taipei Medical University, Taipei City, Taiwan; 3 Department of Pharmacology, School of Medicine, College of Medicine, Taipei Medical University, Taipei City, Taiwan; 4 School of Medicine, College of Medicine, Fu-Jen Catholic University, New Taipei City, Taiwan; 5 School of Public Health, National Defense Medical Center, Taipei City, Taiwan; 6 Department of Medical Research, Cathay General Hospital, Taipei City, Taiwan; 7 Department of Nursing, Yuanpei University of Medical Technology, Hsinchu County, Taiwan; 8 Department of Public Health, College of Medicine, Fu-Jen Catholic University, New Taipei City, 24205 Taiwan; 9 Data Science Center, College of Medicine, Fu-Jen Catholic University, New Taipei City, Taiwan

**Keywords:** Diseases, Neurology

## Abstract

Most proton pump inhibitors (PPIs) inhibit the bioactivation of clopidogrel to its active metabolite. There is controversy concerning whether PPIs alter the effectiveness of clopidogrel in reducing the risk of ischemic stroke (IS). We therefore aimed to examine the risk of IS associated with concomitant use of clopidogrel and omeprazole, a PPI commonly used in clinical settings. We conducted a retrospective cohort study using the National Health Insurance Research Database of Taiwan dated from 2000 to 2013. The study cohorts comprised 407 patients diagnosed with acute coronary syndrome (ACS) and with concomitant use of clopidogrel and omeprazole (the exposed cohort), 814 ACS patients with single use of clopidogrel (the comparison cohort), and 230 ACS patients with concurrent use of clopidogrel and pantoprazole (the reference cohort). The primary outcome was incident IS. The hazard ratios (HRs) and 95% confidence intervals (CIs) derived from the time-dependent Cox regression model were used to assess the association between concomitant use of clopidogrel and omeprazole and the risk of IS. The incidence rate of IS was significantly higher in the exposed cohort (81.67 per 1000 person-years) than in the comparison cohort (57.45 per 1000 person-years), resulting in an adjusted HR of 1.39 (95% CI 1.03–1.74). By contrast, there was no significant difference in the risk of IS between the exposed and reference cohorts (adjusted HR 1.11; 95% CI 0.81–1.52). The present study revealed that patients taking both clopidogrel and omeprazole was associated with an increased risk of IS.

## Introduction

**Figure 1:**
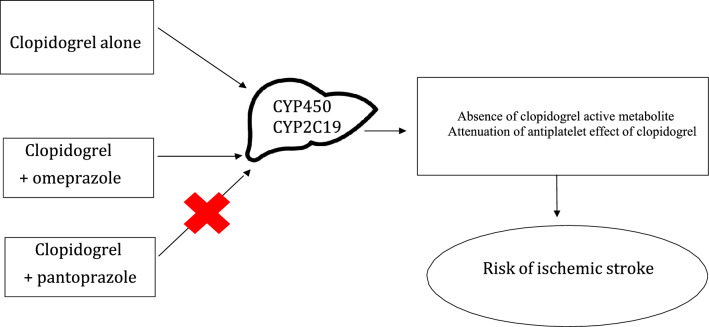
Illustration of mechanisms for the study hypothesis that co-prescription of clopidogrel and Omeprazole could increase the risk of ischemic stroke.

The global burden of stroke is increasing dramatically because of population aging. The Global Burden of Diseases, Injuries, and Risk Factors Study (GBD) showed that stroke was the third-leading cause of death and disability combined and the second-leading cause of death in the world in 2017^1^. Antiplatelet medications, such as aspirin and clopidogrel, have been proven to be beneficial in reducing the recurrent thrombotic events in patients with stroke^2,3^. Clopidogrel is an antiplatelet drug often given with low dose aspirin to patients with acute coronary syndrome or after ischemic stroke (IS), with the aim of preventing further vascular events. Clopidogrel is a prodrug that requires biotransformation into an active metabolite by the hepatic multiple cytochrome (CYP) P450 isoenzymes^13–15^. CYP2C19 is an enzyme contributing to 45% of the metabolism of clopidogrel to an inactive intermediate, and to 21% of the conversion of the intermediate to the active form^15^. As clopidogrel can increase the risk of bleeding, proton pump inhibitors (PPIs) are commonly prescribed with clopidogrel to reduce the risk of serious gastrointestinal bleeding^4^. Even though the administration of PPIs in patients treated with clopidogrel reduces the risk for gastrointestinal bleeding, some pharmacodynamic studies suggested that the antiplatelet effect of clopidogrel is also attenuated by PPIs^9,16–20^ This interaction is due to the inhibition by PPIs of the CYP2C19, which converts clopidogrel to its active metabolite (Fig. 1)^21^. Notably, PPIs differ in their ability to inhibit CYP2C19, omeprazole being a more potent inhibitor than the other members of the class^22–24^. However, there has been much debate about whether some or all PPIs might reduce the effectiveness of clopidogrel^5–12^. Accordingly, some studies showed that omeprazole attenuates the antiplatelet effect of clopidogrel^15–17^ but others did not confirm these findings^18,19^. In contrast, esomeprazole, lansoprazole, pantoprazole and rabeprazole did not affect platelet function in patients treated with clopidogrel^13,15,25–28^. These findings suggest that there is a potentially important pharmacokinetics between clopidogrel and individual PPIs when used at therapeutic doses. Of note, there are limited large-scale studies in Asians, who may have a greater prevalence of CYP2C19 loss-of-function polymorphisms^29^. Indeed, observational studies have shown inhibition of the antiplatelet effect of clopidogrel by PPIs, omeprazole most consistently, which could have significant clinical effects^9,16–20^. However, a number of other observational studies did not show an interaction between clopidogrel and PPIs^18,19^. These conflicting findings highlight the need for large and population-based studies. Given the inconsistent data regarding a possible pharmacological interaction between clopidogrel and PPIs, we sought to examine the risk of IS associated with clinical use of clopidogrel alone or co-prescription of clopidogrel and omeprazole in patients with acute coronary syndrome (ACS) using a nationwide medical claims dataset-National Health Insurance Research Database (NHIRD) in Taiwan.

## Methods and materials

### Ethics statement

Since the data set was released for research purposes and included only scrambled information on patient identification, the study was exempt from informed consent from the subjects. Nevertheless, this study conformed to the Declaration of Helsinki. All methods were performed in accordance with the relevant guidelines and regulations. The study protocol has been approved by the Institutional Review Board of Cathay General Hospital in Taipei City (CGH-P107074).

### Data source

The present study was a retrospective cohort study using medical claims data from the NHIRD. The single-payer comprehensive national health insurance (NHI) program has been at the core of the health care system in Taiwan since 1995^19^. Over the years, more than 99% of the total Taiwanese population has been enrolled in this program^20^. The NHIRD contains comprehensive medical care process information, including demographic data of insured individuals, data of clinical visits, diagnostic codes, and prescription details. Patients’ diagnoses in the database were encoded using the International Classification of Diseases, Ninth revision, Clinical Modification (ICD-9-CM). NHIRD has been used for high quality epidemiological studies with good validity on data regarding diagnoses, drug prescriptions, and hospitalizations^21–24^. The data of this study was obtained from the Longitudinal Health Insurance Database 2000 (LHID 2000). LHID 2000 is a cohort dataset of original medical claims data that included one million beneficiaries randomly sampled from the registry of NHIRD dated between January 1, 2000 and December 31, 2013. There was no significant difference in the distributions of age, sex, and medical care costs between beneficiaries in LHID and all enrollees in NHIRD^19^.

### Study subjects

**Figure 2:**
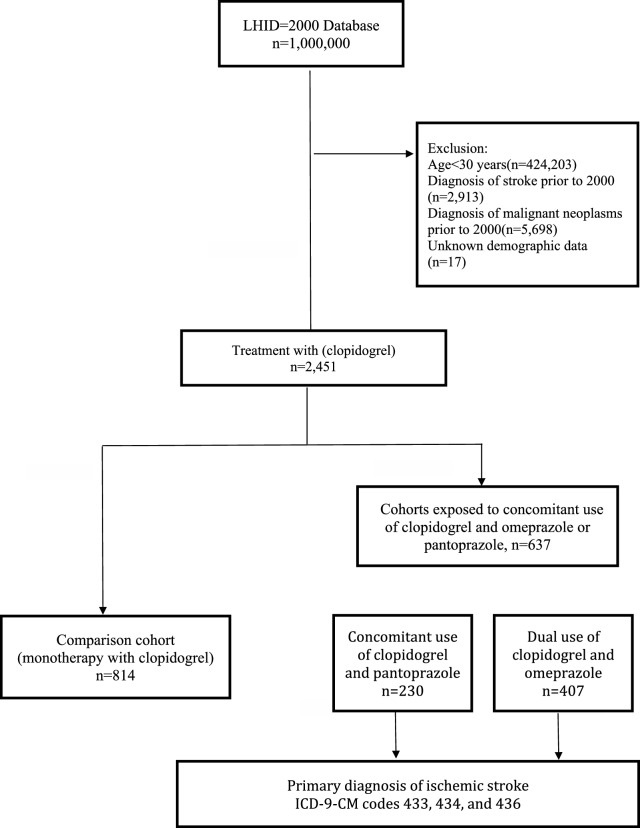
Flowchart of the study sample selection. LHID, longitudinal health insurance database.

The cohort included NHI enrollees 30 years of age or older who were primary hospitalized for ACS between January 1, 2000 and December 31, 2006 (the study period). Prior to the qualifying hospitalization, cohort members had to have ≧ 365 days of NHI enrollment. Among cohort members, we then identified ACS patients first concomitantly exposed to clopidogrel [Anatomical Therapeutic Chemical (ATC) code: B01AC] and omeprazole (ATC code: A02BC01) during the study period as the exposed cohort. Meanwhile, ACS patients initially underwent clopidogrel alone treatment were recruited as the comparison cohort. In addition, pantoprazole was selected as the reference PPI, as it is not a potent inhibitor of CYP2C19 and is considered to have a low potential for drug-drug interactions with clopidogrel^25^. Thus, patients with concomitant use of clopidogrel and pantoprazole (ATC code: A02BC02) were defined as the reference cohort. Cohort members were excluded from the study if < 30 years (n = 424,203), diagnoses of stroke (n = 2913) or malignant neoplasms prior to the cohort entry date (n = 5698), or unknown demographic data (n = 17). In total, there were 407 ACS patients with concomitant use of clopidogrel and omeprazole as the exposed cohort, 814 ACS patients underwent clopidogrel alone treatment as the comparison cohort, and 230 patients with dual use of clopidogrel and pantoprazole as the reference cohort. Follow-up began upon cohort entry and continued until the first occurrence of the following: IS (outcome of interest), death (as assessed by the disenrollment from NHI), or the end of follow-up on December 31, 2013, whichever came first (Fig. 2).

### Ascertainment of IS

We identified cohort members who had a diagnosis of IS (ICD-9-CM codes: 433, 434, and 436) for the first time between January 1, 2007 and December 31, 2013 as the primary outcome. A prior study validating the diagnosis of acute IS in NHI claims data using the Taiwan Stroke Registry as a reference revealed a positive predictive value of 88.4% and sensitivity of 97.3%^26^. We only enrolled patients receiving brain computed tomography or magnetic resonance imaging during hospitalization with the assumption that all patients with symptoms of acute stroke should receive brain imaging. This approach was intended to exclude stroke patients who were hospitalized for rehabilitation during chronic stage. Although brain imaging, especially computed tomography, may not be able to reveal the cerebral infarction in patients with symptoms or signs of acute stroke, it can easily exclude intracranial hemorrhage. Thus, NHIRD had a low probability of misclassifying hemorrhagic stroke into IS if brain imaging was done.

### Covariate assessment and adjustment

In the present study, patients’ demographics, including age and sex, comorbidities, and co-medication prescriptions were identified as covariates. These covariates were included in the regression models for adjustment. We used inpatient and outpatient files to ascertain whether cohort members had comorbidities during the study period, including hypertension, diabetes mellitus, hyperlipidemia, chronic obstructive pulmonary disease, alcoholism or alcohol-related disorders, and tobacco dependence. Comorbidities were determined in a patient if he or she was diagnosed for any aforementioned diseases on at least two outpatient claims or one inpatient claim during the study period. Co-medication prescriptions included anti-hypertensive medications, hypoglycemic agents, statins, and low-dose aspirins. In the current study, we used comorbidities of chronic obstructive pulmonary disease and tobacco dependence as a proxy measure of smoking, alcoholism and alcohol-related disorders as substitute variables of alcohol consumption, and hypertension, diabetes mellitus, and hyperlipidemia as surrogate indicators of obesity.

### Statistical analysis

Chi-square test was used to evaluate the distributions of categorical variables between the study cohorts. Incidence rates of IS were calculated by dividing the number of incident cases of ischemic stroke by the number of person-years of observation in the study cohorts. In addition, the cumulative risks of IS were calculated with the Kaplan–Meier method and compared by log-rank test. Because the exposure of interest in the study cohorts is time-dependent and non-proportional hazards of IS exist in the study cohorts, as indicated by the Schoenfeld global test (*p* = 0.036)^27^, the time-dependent cox proportional hazards regression model were used to compute the hazard ratios (HRs) and accompanying 95% confidence intervals (CIs) to determine the associations of the risk of IS with concomitant use of clopidogrel and omeprazole. Furthermore, we performed stratified analyses on the basis of age and sex to evaluate the consistency of the relationship of the risk of IS with concomitant use of clopidogrel and omeprazole. All statistical tests were two-sided and all *p*-value < 0.05 was considered to be statistically significant. All data alalyses were performed using SAS software, version 9.1 (SAS Institute, Cary, NC).

## Results

The baseline characteristics of the study cohorts, including concomitant use of clopidogrel and omeprazole (the exposed cohort), co-prescription of clopidogrel and pantoprazole (the reference cohort), and single use of clopidogrel (the comparison cohort), are provided in Table 1. There were significant differences in the distributions of sex, age, prevalence of hyperlipidemia, and proportions of taking anti-hypertensive agents, statins, and low-dose aspirins among study cohorts. Basically, the reference cohort of patients with co-prescription of clopidogrel and pantoprazole had higher proportions of females, older age, hyperlipidemia, use of antihypertensive agents, statins, and low-dose aspirins than patients in the other two cohorts.

**Table 1:** Baseline characteristics of study cohorts with concomitant use of clopidogrel and omeprazole (the exposed cohort) and clopidogrel and pantoprazole (the reference cohort) as well as single use of clopidogrel (the comparison cohort).

Variable	Exposed cohort	Comparison cohort	Reference cohort	*P*-value
	n = 407	n = 814	n = 230	
Sex				0.021
Male	65.60	65.60	61.74	
Female	34.40	34.40	38.26	
Age, years				< .0001
30–49	38.08	38.08	28.26	
50–69	43.00	43.00	48.26	
> 70	18.92	18.92	23.48	
Comorbidities				
Hypertension	78.87	78.75	81.74	0.598
Diabetes mellitus	41.28	37.71	43.91	0.176
Hyperlipidemia	31.94	46.44	56.52	< .0001
COPD	66.58	64.62	69.13	0.417
Tobacco dependence	1.23	2.58	3.91	0.094
ARD	0.49	0	0	0.076
Obesity	0	0.25	0.43	0.476
Use of co-medications (%)				
Anti-hypertensive agents	27.27	29.36	38.26	0.011
Hypoglycemic agents	22.60	20.02	23.91	0.344
Statins	38.57	50.12	58.26	< .0001
Low-dose aspirins	23.59	42.14	42.17	< .0001

The exposed and comparison cohorts were matched on age and sex.

COPD, chronic obstructive pulmonary disease; ARD, alcohol-related disorders.

**Figure 3:**
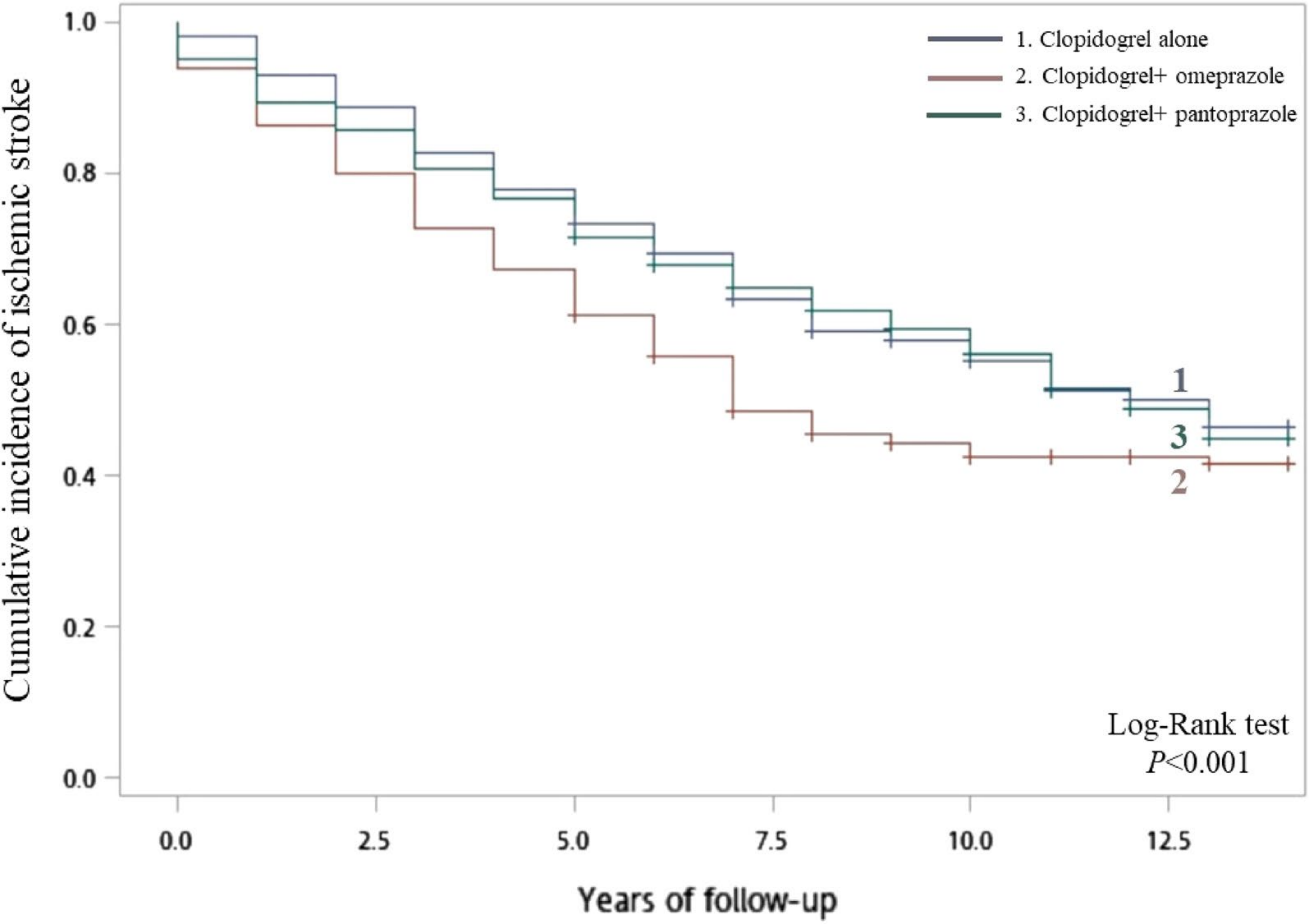
Kaplan–Meier curves for the cumulative risk of ischemic stroke among patients with comcomitant use of clopidogrel and omeprazole and those with single use of clopidogrel.

Results in Table 2 show that incidence rate of IS was significantly higher in patients with concomitant use of clopidogrel and omeprazole (81.67 per 1000 person-years) than in patients with use of clopidogrel alone (57.45 per 1000 person-years). After adjustment for potential confounders including age, sex, comorbidities (hypertension, diabetes mellitus, hyperlipidemia, chronic obstructive pulmonary disease, tobacco dependence, alcohol-related disorders), and use of co-medications (anti-hypertensive drugs, hypoglycemic agents, statins, and low-dose aspirins), there was a significant HR of 1.65 (95% CI 1.12–2.86) for the association between concomitant use of clopidogrel and omeprazole and risk of IS. By contrast, there was no significant difference in the incidence rate of IS between the reference cohort (61.00 per 1000 person-years) and the comparison cohort with an adjusted HR of 1.13 and 95% CI of 0.81–3.53. According to the Kaplan–Meier analysis, the cumulative incidence of IS was significantly different among study cohorts (log-rank test, *p* < 0.001). The exposed cohort of patients with concomitant use of clopidogrel and omeprazole had a higher cumulative incidence of IS than patients in the other two cohorts (Fig. 3).

**Table 2:** Risk of ischemic stroke associated with concomitant use of clopidogrel and omeprazole and co-prescription of clopidogrel and pantoprazole.

Variable	No. of patients	No. of PYs	No. of ischemic stroke	Incidence rate (per 1000 PYs)	Adjusted HR (95% CI)
Clopidogrel alone	814	6370.28	366	57.45	1.00 (reference)
Clopidogrel + omeprazole	407	2779.59	227	81.67	1.65 (1.12–2.86)
Clopidogrel + pantoprazole	230	1737.61	106	61.00	1.13 (0.81–3.53)

PYs, person-years; HR, hazard ratio; CI, confidence interval.

Hazard ratios were adjusted for age, sex, comorbidities, including hypertension, diabetes mellitus, hyperlipidemia, chronic obstructive pulmonary disease, tobacco dependence, alcohol-related disorders, as well as use of co-medications, including anti-hypertensive drugs, hypoglycemic agents, statins, and low-dose aspirins.

Results of the association between duration of receiving clopidogrel and omeprazole prescriptions and the risk of IS are presented in Table 3. Patients with concomitant use of clopidogrel and omeprazole for 4–7 years (T_2_) had a higher risk of IS than those with dual use of clopidogrel and omeprazole for < 4 years (T_1_), resulting in a HR of 1.21 (95% CI 0.76–2.42). In addition, patients with concomitant use of clopidogrel and omeprazole for > 7 years (T_3_) also had a higher risk of IS than those with concurrent use of clopidogrel and omeprazole for < 4 years (T_1_), resulting in a HR of 1.64 (95% CI 0.21–3.24). There appeared to be a duration-dependent risk of IS (*p* for trend = 0.057). That is, the risk of IS increased with extending duration of concomitant use of clopidogrel and omeprazole.

**Table 3:** Association between duration of receiving clopideogrel and omeprazole prescriptions and risk of ischemic stroke.

Duration of prescription	No. of patients	No. of PYs	No. of ischemic stroke	Incidence rate (per 1000 PYs)	Adjusted HR (95% CI)
T_1_ (< 4 years)	606	4660.14	266	57.08	1.00 (reference)
T_2_ (4–7 years)	371	2860.41	201	70.27	1.21 (0.76–2.42)
T_3_ (> 7 years)	244	1327.36	126	94.92	1.64 (0.21–3.24)

PYs, person-years; HR, hazard ratio; CI, confidence interval.

Hazard ratios were adjusted for age, sex, comorbidities, including hypertension, diabetes mellitus, hyperlipidemia, chronic obstructive pulmonary disease, tobacco dependence, alcohol-related disorders, as well as use of co-medications, including anti-hypertensive drugs, hypoglycemic agents, statins, and low-dose aspirins.

The stratified analyses based on subgroups formed by sex and age showed consistent results with the primary findings that concomitant use of clopidogrel and omeprazole was associated with an increased risk of IS as compared with use of clopidogrel alone. By contrast, there was no significant relationship of concomitant use of clopidogrel and pantoprazole with the risk of IS (Table 4).

**Table 4:** Risk of ischemic stroke in relation to concomitant use of clopidogrel and omeprazole stratified by sex and age.

Variable	No. of patients	No. of PYs	No. of ischemic stroke	Incidence rate (per 1000 PYs)	Adjusted HR (95% CI
Men					
Clopidogrel alone	468	5572.04	203	36.43	1.00 (reference)
Clopidogrel + omeprazole	267	1714.19	140	81.67	1.41 (1.02–2.96)
Clopidogrel + pantoprazole	142	1042.57	70	67.14	1.12 (0.82–2.81)
Women					
Clopidogrel alone	346	2707.76	163	60.20	1.00 (reference)
Clopidogrel + omeprazole	140	956.12	87	90.99	1.69 (1.06–2.84)
Clopidogrel + pantoprazole	88	664.82	36	54.15	1.06 (0.58–2.75)
Age 30–59 years					
Clopidogrel alone	274	2144.29	43	20.05	1.00 (reference)
Clopidogrel + omeprazole	137	935.64	70	74.81	2.35 (1.52–3.61)
Clopidogrel + pantoprazole	122	921.69	87	94.39	1.21 (0.81–2.18)
Age ≥ 60 years					
Clopidogrel alone	540	4225.98	279	66.02	1.00 (reference)
Clopidogrel + omeprazole	270	1843.95	157	85.14	1.48 (0.97–3.76)
Clopidogrel + pantoprazole	108	815.92	63	77.21	1.12 (0.80–2.41)

PYs, person-years; HR, hazard ratio; CI, confidence interval.

Hazard ratios were adjusted for age, sex, comorbidities, including hypertension, diabetes mellitus, hyperlipidemia, chronic obstructive pulmonary disease, tobacco dependence, alcohol-related disorders, as well as use of co-medications, including anti-hypertensive drugs, hypoglycemic agents, statins, and low-dose aspirins.

Results of multivariable analysis of the risk of IS are shown in Table 5. After adjustment for potential confounders, patients with concomitant use of clopidogrel and omeprazole had significantly higher risk of developing IS than those with single use of omeprazole (adjusted HR 1.39; 95% CI 1.03–1.74).

**Table 5:** Multivariable analysis of the risk factors associated with ischemic stroke.

Variable	No. of patients	No. of PYs	No. of ischemic stroke	Incidence rate (per 1,000 PYS)	Adjusted HR (95% CI)
Sex					
Women	574	4121.32	286	69.40	1.0
Men	877	6516.11	413	63.38	0.89 (0.71–1.13)
Age					
30–49	296	2439.04	111	45.51	1.0
50–69	636	4750.92	288	60.62	1.13 (0.82–1.55)
≧70	519	3451.35	300	86.92	1.62 (1.18–2.23)
Hyperlipidemia					
No	813	5902.38	394	66.75	1.0
Yes	638	4740.34	305	64.34	1.04 (0.82–1.32)
Use of low-dose aspirns					
No	915	6496.50	426	65.57	1.0
Yes	536	4143.28	273	65.89	1.18 (0.86–1.64)
Prescriptions					
Clopidogrel alone	814	6105.00	366	59.95	1.0
Clopidogrel + omeprazole	407	2739.11	227	86.52	1.39 (1.03–1.74)
Clopidogrel + pantoprazole	230	1800.90	106	58.86	1.11 (0.81–1.52

PYs, person-years; HR, hazard ratio; CI, confidence interval.

## Discussion

This nationwide cohort study based on medical claims data made available by Taiwan’s NHIRD demonstrated that patients with concomitant use of clopidogrel and omeprazole had an increased risk of IS as compared with those with use of clopidogrel alone. This positive association was consistently across sex and age categories.

Platelet activation and aggregation are key elements of the pathogenesis of IS. Drugs that impair platelet function are an important part of treatment for patients with IS. Clopidogrel is a prodrug that is converted in the liver to an active thiol metabolite, which irreversibly inhibits the platelet P2Y12 adenosine diphosphate receptor^30^. This bioactivation is mediated by hepatic cytochrome P450 isoenzymes, with cytochrome P450 2C19 playing a major role^14^. Patients with loss-of-function polymorphisms of P450 2C19 have lower levels of the active metabolite of clopidogrel, diminished platelet inhibition during clopidogrel treatment and an increased risk of cardiovascular events relative to those without such polymorphisms^14^. Given the importance of cytochrome P450 2C19 in the bioactivation of clopidogrel, drugs that inhibit this enzyme may reduce the antiplatelet effect of clopidogrel. Emerging evidence suggests that some PPIs can inhibit cytochrome P450 2C19, possibly altering clopidogrel’s pharmacokinetics and potentially leading to an increased risk of adverse cardiac outcomes^5,14,31^. It has been noted that PPIs differ in their ability to inhibit CYP2C19, omeprazole being a more potent inhibitor than the other members of the class^22–24^. Indeed, among high-risk angioplasty patients treated with acetylsalicylic acid and clopidogrel, use of omeprazole significantly reduced the antiplatelet activity of clopidogrel^10^. Likewise, the present study indicated that patients received both clopidogrel and omeprazole had a significantly elevated risk of IS as compared with those received clopidogrel alone. To test the specificity of our primary findings, the current study synchronously examined concomitant use of clopidogrel and pantoprazole as a tracer exposure. The drug pantoprazole has clinical indications similar to omeprazole, yet pantoprazole is not a potent inhibitor of CYP2C19. The results showed that there was no significant relationship of concomitant use of clopidogrel and pantoprazole with the risk of IS. Although there is clear evidence of clinical interaction between PPIs and platelet antiaggregants clopidogrel from the pharmacological point of view, the bibliography consulted shows contradictory results. Nevertheless, our finding of increased IS risk associated with concomitant use of clopidogrel and omeprazole is consistent with in vitro findings suggesting that there are muti-fold differences in the strength of CYP2C19 inhibition by omeprazole^31^.

The strengths of this study include the use of a nationwide population-based comprehensive prescription database rather than self-reported records (thereby minimizing recall bias). In addition, the NHIRD covers a highly representative sample of Taiwan’s general population because the reimbursement policy is universal and operated by a single-payer. This allowed us to perform our analysis in a real-life setting in an unselected patient population. Furthermore, our cohort study had the advantage of collecting information from population-based databases with prospectively registered and virtually completed data on drug prescriptions, thus minimizing information biases. However, the results of this study need to be interpreted mindful of several methodological limitations. It has been noted that studies based on medical claims data are often biased because the information on potential confounders contained in claims dataset is often limited^32^. There were no data on important IS risk factors such as smoking status, blood pressure, and obesity, in the NHIRD. In addition, claims data in the NHIRD lacked laboratory parameters. Accordingly, we were unable to examine lipoprotein profiles and genetic polymorphisms in CYP enzymes. It is likely that our findings could subject to residual confounding. Furthermore, medication exposure, determined from computerized records of dispensed prescriptions in the NHIRD did not reflect adherence, actual doses used, or over-the-counter medication use and thus is subjected to misclassification that most probably would bias to the null^33^.

## Conclusions

In summary, this study found that among patients taking both clopidogrel and a PPI omeprazole that inhibits cytochrome P450 2C19 was associated with an increased risk of IS. Data from additional studies will be important to clarify the clinical implications of the current study.

## Data Availability

Te data sets used in the present study are not available based on the policy of using nation-wide insurance claims datasets by the Ministry of Health and Welfare in Taiwan. Correspondence and requests for materials should be addressed to Chien-An Sun.
